# Artificial Bee Colony Algorithm for Solving Optimal Power Flow Problem

**DOI:** 10.1155/2013/159040

**Published:** 2013-12-29

**Authors:** Luong Le Dinh, Dieu Vo Ngoc, Pandian Vasant

**Affiliations:** ^1^Faculty of Mechanical, Electrical and Electronic Engineering, HCMC University of Technology (HUTECH), Ho Chi Minh City, Vietnam; ^2^Department of Power Systems, HCMC University of Technology (HCMUT), Ho Chi Minh City, Vietnam; ^3^University Technology Petronas, 31750 Tronoh, Perak, Malaysia

## Abstract

This paper proposes an artificial bee colony (ABC) algorithm for solving optimal power flow (OPF) problem. The objective of the OPF problem is to minimize total cost of thermal units while satisfying the unit and system constraints such as generator capacity limits, power balance, line flow limits, bus voltages limits, and transformer tap settings limits. The ABC algorithm is an optimization method inspired from the foraging behavior of honey bees. The proposed algorithm has been tested on the IEEE 30-bus, 57-bus, and 118-bus systems. The numerical results have indicated that the proposed algorithm can find high quality solution for the problem in a fast manner via the result comparisons with other methods in the literature. Therefore, the proposed ABC algorithm can be a favorable method for solving the OPF problem.

## 1. Introduction

Optimal power flow (OPF) problems are the important fundamental issues in power system operation. In essence, they are the optimization problems and their main objective is to reduce the total generation cost of units while satisfying various constraints in the system. The OPF problem has been widely used in power system operation and planning [1]. Many techniques such as linear programming (LP) [2–4], nonlinear programming (NLP) [5–8], and quadratic programming (QP) [9] have been applied for solving the OPF problem. For implementation of these methods, the problem needs to be convexity and thus there is a fore to simplify the problem to ensure the convexity. However, the OPF is in general a nonconvex problem with many local minima. In addition, the classical optimization methods are highly sensitive to the starting points and frequently converge to local optimum solution or diverge altogether.

The LP methods are fast and reliable for solving optimization problems but the main disadvantage of the LP methods is that both objective and constraints of the problem need to be piecewise linear functions. The NLP methods suffer another problem of convergence and algorithmic complexity. Newton-based algorithm [10] suffers difficulties in handling large number of inequality constraints. Moreover, this method has also a drawback the convergence characteristics sensitive to the initial conditions. Interior point (IP) methods [11–13] convert the inequality constraints of the problem to equality ones by adding slack variables. In the IP method, if the step size is not properly chosen, the sublinear problem may lead to an infeasible solution in the original nonlinear domain [12]. These methods are usually confined to specific cases of the OPF problem and do not offer great freedom in objective functions or the type of constraints that may be used. Therefore, it is important to develop new and more general and reliable algorithms for dealing with the nonlinear OPF problem. Metaheuristic search techniques such as genetic algorithm (GA) [14–16], gradient projection method [17], and evolutionary programming (EP) [18] have been implemented for solving the OPF problem. However, the recent findings have identified some deficiencies in the GA performance. Recently, a new evolutionary computation technique, called artificial bee colony (ABC), has been proposed and introduced by Karaboga in [19]. The ABC algorithm is developed based on inspecting the behaviors of real bees on finding nectar and sharing the information of food sources to the bees in their hive. The main advantages of the ABC algorithm over other optimization methods for solving optimization are simplicity, high flexibility, strong robustness [20, 21], few control parameters [22], ease of combination with other methods [21], ability to handle the objective with stochastic nature [23], fast convergence, and both exploration and exploitation.

In this paper, the ABC algorithm is proposed for solving OPF problem with both continuous and discrete control variables. The continuous controllable system quantities are generator MW, controlled voltage magnitude, and switchable shunt devices while the discrete ones are transformer tap settings. The objective is to minimize the total fuel cost of thermal generators by optimizing the control variables within their limits so that they cause no violation on other quantities (e.g., transmission-circuit loading, load bus voltage magnitude, and generator MVAR) occurring in either the normal or outage case of system operating conditions. The proposed algorithm has been tested on the IEEE 30-bus, 57-bus, and 118-bus systems for both normal and contingent cases. The obtained results from the proposed method are compared to those from EP [18], particle swarm optimization (PSO) [24], ant colony optimization (ACO) [25], self-adaptive DE (SADE_ALM) [26], modified differential evolution (MDE) [27], improved EP (IEP) [28], QN and ACO methods in [29], hybrid GA (HGA) [30], and improved PSO (IPSO) [31].

## 2. Optimal Power Flow Problem Formulation

The OPF problem can be described as an optimization (minimization) process with nonlinear objective function and nonlinear constraints. In general, the general OPF problem can be expressed as follows:

(1) minimize  F(x)subject  to g(x)=0       h(x)≤0,

where *F*(*x*) is the objective function, *g*(*x*) represents the equality constraints, *h*(*x*) represents the inequality constraints, and *x* is the vector of the control variables varied by a control center operator (generated active and reactive powers, generation bus voltage magnitudes, transformer taps, etc.).

The essence of the OPF problem resides in minimizing the objective function and simultaneously satisfying the load flow equations (equality constraints) without violating the inequality constraints. The fuel cost function for thermal generators is given by

(2) $$\begin{array}{c} F\left(x\right)=\sum_{i-1}^{N_{G}}\left(a_{i}+b_{i}P_{Gi}+c_{i}P_{Gi}^{2}\right), \end{array}$$

where *N*_*G*_ is the number of generation buses including the slack bus, *P*_*G*_ is the generated active power at bus *I*, and *a*_*i*_, *b*_*i*_, and *c*_*i*_ are the unit costs curve for *i*th generator.

The smooth quadratic fuel cost function of the generating units is given by (2), where the valve point effects are neglected. The generating units with multivalve steam turbines exhibit a greater variation in the fuel-cost function. Since the valve point results in the ripples, a cost function contains higher order nonlinearity. Therefore, (2) should be replaced by (3) for taking into consideration the valve point effects. The sinusoidal function is thus added to the quadratic cost functions as follows [32]:

(3) $$\begin{array}{c} F_{i}\left(P_{i}\right)=a_{i}+b_{i}P_{i}+c_{i}P_{i}^{2}+\left|e_{i}\times \sin\left(f_{i}\times \left(P_{i,\min}-P_{i}\right)\right)\right|, \end{array}$$

where *e*_*i*_ and *f*_*i*_ are the fuel cost coefficients of *i*th unit with valve point effects.

While minimizing the fuel cost function, it is necessary to make sure that the generation still supplies the load demands plus losses in transmission lines. Usually, the power flow equations are used as equality constraints [33].

Consider the following:

(4) $$\begin{array}{c} \left[\begin{array}{c} \Delta P_{i}\\ \Delta Q_{i} \end{array}\right]=\left[\begin{array}{c} P_{i}\left(V,\theta \right)-\left(P_{G_{i}}-P_{D_{i}}\right)\\ Q_{i}\left(V,\theta \right)-\left(Q_{G_{i}}-Q_{D_{i}}\right) \end{array}\right]=0, \end{array}$$

where the active and reactive power injection at bus *i* are defined in the following equation:

(5) $$\begin{array}{c} P_{i}\left(V,\theta \right)=\sum_{j=1}^{N_{B}}V_{i}V_{j}\left(G_{ij}\cos\theta_{ij}+B_{ij}\sin\theta_{ij}\right),\\ Q_{i}\left(V,\theta \right)=\sum_{j=1}^{N_{B}}V_{i}V_{j}\left(G_{ij}\sin\theta_{ij}+\cos B_{ij}\theta_{ij}\right). \end{array}$$

The inequality constraints of the OPF problem reflect the limits on physical devices in power systems and they also ensure the system security. The usual types of inequality constraints are upper bus voltage limits at generations and load buses, lower bus voltage limits at load buses, reactive power limits at generation buses, maximum active power limits corresponding to lower limits at some generators, maximum line loading limits, and limits on tap settings. The inequality constraints of the problem include the following.

*Generation Constraint*. Generator voltages, real power outputs, and reactive power outputs are restricted by their upper and lower bounds as follows:

(6) $$\begin{array}{c} P_{Gi,\min}\leq P_{Gi}\leq P_{Gi,\max}\;\text{for}\;i=1,2,\ldots ,N_{G},\\ Q_{Gi,\min}\leq Q_{Gi}\leq Q_{Gi,\max}\;\text{for}\;i=1,2,\ldots ,N_{G},\\ V_{Gi,\min}\leq V_{Gi}\leq V_{Gi,\max}\;\text{for}\;i=1,2,\ldots ,N_{G}. \end{array}$$

*Shunt VAR Constraint*. Shunt VAR compensations are restricted by their upper and lower bounds as follows:

(7) $$\begin{array}{c} Q_{Ci,\min}\leq Q_{Ci}\leq Q_{Ci,\max}\;\text{for}\;i=1,2,\ldots ,N_{C}, \end{array}$$

where *N*_*C*_ is the number of shunt compensators.

*Transformer Tap Constraint.* Transformer tap settings are restricted by their upper and lower bounds as follows:

(8) $$\begin{array}{c} T_{i,\min}\leq T_{i}\leq T_{i,\max}\;\text{for}\;i=1,2,\ldots ,N_{T}, \end{array}$$

where *N*_*T*_ is the number of transformers with tap. 

*Security Constraint.* Voltages at load bus are restricted by their upper and lower bounds as follows:

(9) $$\begin{array}{c} V_{Li,\min}\leq V_{Li}\leq V_{Li,\max}\;\text{for}\;i=1,2,\ldots ,N_{L}, \end{array}$$

where *N*_*L*_ is the number of load buses.

## 3. Artificial Bee Colony Algorithm for OPF Problem

### 3.1. Artificial Bee Colony Algorithm

Artificial bee colony (ABC) algorithm is introduced by Karaboga in 2005 [19]. The ABC algorithm was formed by observing the activities and behavior of the real bees while they were looking for the nectar resources and sharing the amount of the resources with the other bees.

Data flow creation around the beehive is a behavior of bees which involves the fundamentals of the swarm intelligence. The colony of artificial bees consists of three groups of bees including employed bees, onlookers, and scouts. Each type of bees has a different role in the optimization process. The employed bees wait above the nectar source and keep the neighboring sources in memory while the onlooker bees get that data from the employed bees and make a resource choice to collect the nectar and the scouts are very much accountable for calculation. The algorithm for solving an optimization problem is comprised of three steps. In the first step, the employed bees are sent to scamper for resources and the nectar amount is computed. In the second step, the onlooker bees make a resource choice appropriate to the data they took from the new identified nectar resources. Finally, in the last step, one of the employed bees is chosen at random as a scout bee and it is sent to the sources to identify new sources [34]. A half of the bees in the colony are chosen as employed bees and the rest half are chosen as the onlooker bees in the algorithm. Therefore, the number of the employed bees is equal to the number of nectar sources. The food sources in the approach refer to the probable solutions of the issue to be optimized.

In the first step of the ABC algorithm, the initial solutions are randomly created in the particular range of the variables *x*_*i*_  (*i* = 1, 2, 3,…, *S*). Secondly, each employed bee identifies the new sources whose amounts are equal to the half of the total sources. Equation (10) is used to find a new source as follows:

(10) $$\begin{array}{c} V_{ij}=x_{ij}+\varphi_{ij}\left(x_{ij}-x_{kj}\right). \end{array}$$

In (10), *k* ∈ {1, 2,…, *N*} and *j* ∈ {1, 2,…, *D*} are randomly chosen indexes. Although *k* is randomly determined, it has to be different from *i* and *φ*_*ij*_ is a random number between [0, 1]. This parameter controls the production of the neighbor food sources around and represents the *x*_*ij*_ comparison of two food positions visually by a bee. After each candidate source position *V*_*ij*_ is produced and then evaluated by the artificial bee, its performance is compared with its previous one. If the new food has equal or better nectar than the old source, it is used to replace the old one in the memory. Otherwise, the old one is retained in the memory.

In the third step, the onlooker bees select a food source with the probability given in (11).

Consider the following:

(11) $$\begin{array}{c} P_{i}=\frac{\text{fi}{\text{t}}_{i}}{\sum_{j=1}^{S_{N}}\text{fi}{\text{t}}_{j}}, \end{array}$$

where fit_*i*_ is the fitness value of the solution *i* which is proportional to the nectar amount of the food source in the position *i* and *j* is the number of food sources which is equal to the number of employed bees.

The scout bees are very much accountable for random researches in each colony. The scout bees do not use any pre-knowledge and facts when they are looking for nectar sources, thus their research is randomly done completely [35]. The scout bees are chosen among the employed bees with respect to the limit parameters. If a solution that denotes a source is not realized with particular number of trials, then this source is discarded. The bee of that source identifies the new source chosen as a scout bee. The number of incomings and outgoings to a source is obtained by the “limit” parameter. Identifying a new source of a scout bee is given in (12).

Consider the following:

(12) $$\begin{array}{c} X_{ij}=X_{j}^{\min}+\left(X_{j}^{\max}-X_{j}^{\min}\right)*\text{rand}\left(0,1\right), \end{array}$$

where *X*_*j*_^min⁡^ and *X*_*j*_^max⁡^ are the minimum and maximum limits of the parameter to be optimized.

In the ABC algorithm, the stopping criterion is usually based on number of iterations. Normally, stopping criteria of an optimization algorithm are based on maximum number of iterations or maximum error between two consecutive iterations. However, the maximum error cannot be applicable for metaheuristic search methods since they usually suffer unimproved result for several iterations during the convergence process. Therefore, the stopping criterion for the proposed ABC is only based on the maximum number of iterations.

### 3.2. Implementation of ABC for the OPF Problem

The overall algorithm of ABC for solving the OPF problem is as follows.

**Step 1**

Initialize food sources and select parameters for the ABC algorithm.

**Step 2**

Calculate power flow using Newton-Raphson algorithm based on the initial values.

**Step 3**

Calculate fitness function based on the obtained solution of power flow. Set the initial food sources as the best solution. Set the iteration counter to 1.

**Step 4**

Determine position for employed bees.

**Step 5**

Solve power flow problem using Newton-Raphson method based on the position of employed bees.

**Step 6**

Calculate the fitness function corresponding to the new position of the employed bees.

**Step 7**

Compare the new value of the fitness function and the initialized one to obtain the best one.

**Step 8**

If not all onlooker bees are distributed to food sources, update the new position for the onlooker bees and return to Step 5.

**Step 9**

Determine unimproved food sources when the “limit” is exceeded.

**Step 10**

If any unimproved food sources were found, initialize food source for the scout bees, solve power flow problem, and calculate fitness function value.

**Step 11**

Update the best food source position and corresponding fitness function values.

**Step 12**

If the maximum number of iterations is not reached, increase the iteration counter and return to Step 4.

**Figure 1:**
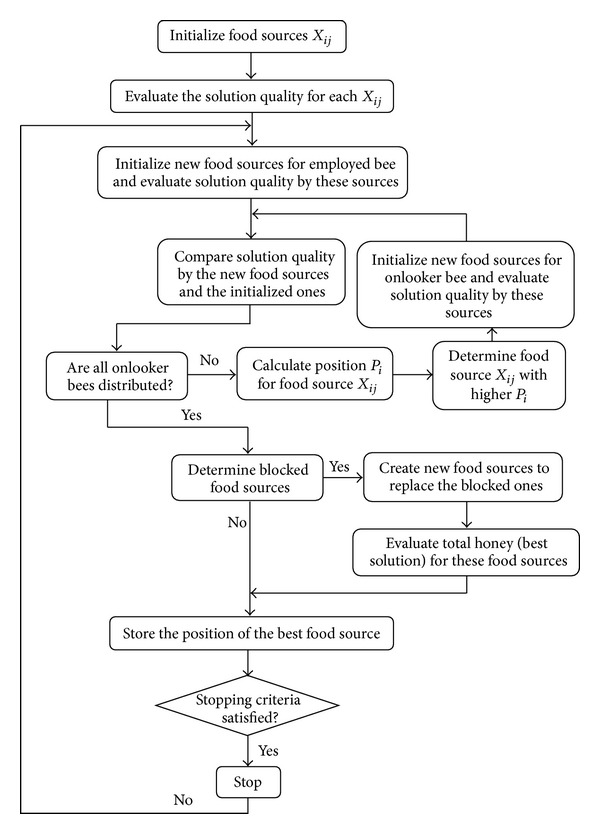
Flowchart of the ABC algorithm.

The flowchart of the ABC algorithm for solving an optimization problem is shown in Figure 1.

## 4. Numerical Results

The proposed ABC algorithm is tested on the IEEE 30-bus, 57-bus, and 118-bus systems and the obtained results are compared to those from other methods available in the literature. The proposed ABC algorithm is coded in Matlab R2009a and run on an Intel Core 2 Duo CPU 1.66 GHz processor with 2.0 GB of RAM. The stopping criterion of the algorithm is the maximum number of iterations. In this paper, the power flow solution for test systems using Newton-Raphson method is obtained from Matpower [36].

For a fair comparison of computational time among the solutions solving the problem using different computers, we calculate floating-point operations (FLOPs) for each method by multiplying floating-point operations per second (FLOPS) with their computational time. FLOPS can be calculated using the equation in [37] as follows:

(13) $$\begin{array}{c} \text{FLOPS}=\text{cores}\times \text{clock}\times \frac{\text{FLOPs}}{\mathrm{cycle}}, \end{array}$$

where core is the number of cores in computer and clock is the processor speed.

For most microprocessors, they can perform 4 FLOPs per clock cycle [37]. Therefore, different processors can be converted into a common base for comparison. In this paper, we choose the computer used for ABC as a base and other computers will be converted into this base. The adjusted CPU time from other methods by other computers will be calculated as follows:

(14) Adjusted  CPU  time(s)  =Given  FLOPSFLOPS  of  ABC×Given  CPU  time(s).

### 4.1. The IEEE 30-Bus System

The IEEE 30-bus system consists of 6 generators, 4 transformers, 41 lines, and 2 shunt reactors. All generator active power, generator bus voltages, and transformer tap setting are considered as continuous for simplicity. The generators cost coefficients of the IEEE 30-bus test system are given in [36].

#### 4.1.1. The OPF Problem with Quadratic Fuel Cost Functions

**Figure 2:**
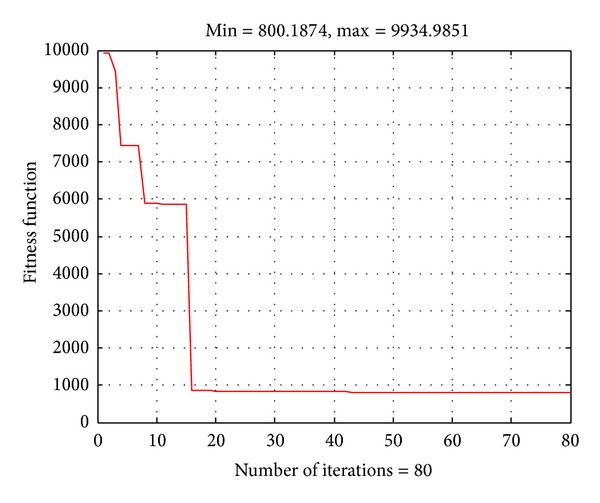
Convergence characteristic of the ABC algorithm for the IEEE 30-bus system with quadratic fuel cost functions.

In this case, the units cost curves are represented by quadratic function. The obtained results using ABC are given in Table 1.  Figure 2 shows the convergence characteristic of the ABC algorithm. As observed, there is no change in the fuel cost function value after 20 iterations. That means the optimal solution for the problem can be obtained within 20 iterations. The obtained total cost and computational time from the ABC algorithm for the system are compared to those from EP [18], PSO [24], and ACO [25] as in Table 1. The result comparison has indicated that the proposed ABC algorithm can obtain better total cost than the others. For the computational time, the proposed ABC is slower than EP but faster than PSO. Therefore, the proposed ABC algorithm is effective for the OPF problem for this system. Note the adjusted computational time in Table 1 is given in the appendix.

#### 4.1.2. The OPF Problem for Units with Valve Point Effects

**Figure 3:**
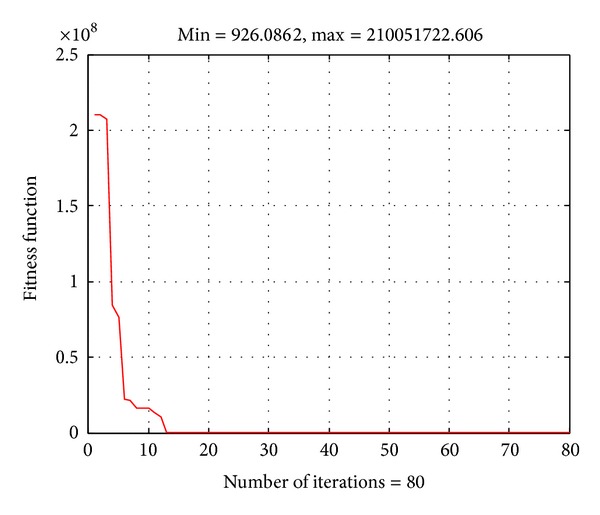
Convergence characteristic of the ABC algorithm for the IEEE 30-bus system with valve point effects.

In this case, the generator fuel cost curves of generator at buses 1 and 2 are represented by a quadratic function with rectified sine components as (3). The generator cost coefficients of those two generators are given in [26]. The obtained results by the proposed ABC algorithm are shown in Table 2 and the convergence characteristic of the proposed method for the system in this case is given in Figure 3. The total cost and computational time obtained by the proposed method are also compared to those from IEP [26], MDE [27], and SADE_ALM [26]. The result comparison has also indicated that the proposed ABC algorithm can find better total cost than the others with a fast computational manner. Therefore, the proposed ABC algorithm is also very effective for solving the problem for this case.

### 4.2. The IEEE-57 Bus-System

**Figure 4:**
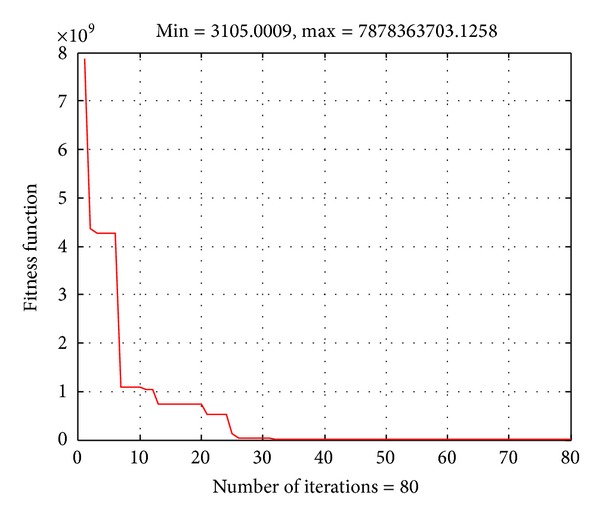
Convergence characteristic of the ABC algorithm for the IEEE 57-bus system.

In this case, the IEEE 57-bus system is considered to investigate the effectiveness of the proposed algorithm. The IEEE 57-bus system consists of 7 generators, 15 transformers, and 80 transmission circuits. The single-line diagram of this system and the detailed data are given in [36]. The total load demand of the system is 1250.8 MW. The obtained result by the proposed method is compared to those from QN and ACO in [29] and HGA [30] methods as shown in Table 3. The convergence characteristic of the proposed algorithm for the system is given in Figure 4. In this case, the number of iterations for the proposed method to obtain the optimal solution is less than 30. The result comparison has also revealed that the proposed method can obtain better total cost than the other methods with faster computational time.

### 4.3. The IEEE-118-Bus System

The proposed ABC algorithm has been tested on large-scale system of 118 buses [38]. The system comprises 54 generators with a total installed capacity of 8190 MW and the system demand is 3668 MW.

**Figure 5:**
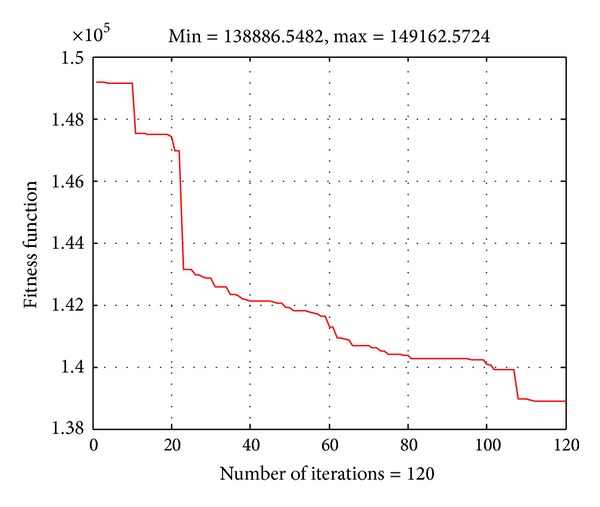
Convergence characteristic of the ABC algorithm for the IEEE 118-bus system.

Table 4 shows the minimum, mean, and maximum cost achieved by the proposed ABC algorithm for 50 runs. The convergence characteristic of the proposed algorithm for the system is given in Figure 5. As observed, the number of iterations for obtaining the result in this case is higher than the previous ones since this a large-scale system and the algorithm needs more time and iterations for obtaining the result. The result comparison in Table 4 has also indicated that the proposed ABC algorithm can obtain better total cost with faster computational time than the IPSO method. Therefore, the proposed ABC algorithm is also very effective for the large-scale systems.

## 5. Conclusion

In this paper, the ABC algorithm is successfully applied for solving the OPF problem. The ABC algorithm is based on the foraging behavior of honey bees for finding global solution for optimization problems. The advantages of the proposed algorithm are robustness, fast calculation, flexibility, and few setting parameters. However, the ABC algorithm also suffers a drawback of search space limited by initial solution. In fact, this drawback can be overcome using normal distribution sample in the initial step. The proposed ABC algorithm has been tested on different systems and the obtained result for each system is compared to other methods in the literature. The result comparisons have shown that the proposed algorithm can obtain better optimal solution than many other methods with a fast computational manner, especially for large-scale systems. Therefore, the proposed ABC algorithm can be a favorable method for solving OPF problems in power systems.

## Figures and Tables

**Table 1:** Result comparison for the IEEE 30-bus system with quadratic fuel cost functions.

	Min	Max	EP [18]	PSO [24]	ACO [25]	ABC
*P*_*g*1_ (MW)	50	200	173.8262	175.6915	177.8635	177.7793
*P*_*g*2_ (MW)	20	80	49.998	48.6390	43.8366	51.3022
*P*_*g*5_ (MW)	15	50	21.386	21.4494	20.8930	21.1685
*P*_*g*8_ (MW)	10	35	22.63	22.7200	23.1231	14.6921
*P*_*g*11_ (MW)	10	30	12.928	12.2302	14.0255	14.6290
*P*_*g*13_ (MW)	12	40	12.000	12.000	13.1199	12.7487
Loss (MW)			9.3683	9.3301	9.4616	8.9197
Min. cost ($/h)			802.5557	802.0136	803.123	800.1874
Avg. cost ($/h)			—	—	—	807.1770
Max. cost ($/h)			—	—	—	826.6428
Std. dev.			—	—	—	3.7156
CPU (s)			51.4	77.672	20	22.73
Adjusted CPU (s)			3.1	56.15	22.53	22.73

**Table 2:** Result comparison for the IEEE-30 bus system with valve point effects.

	Min	Max	IEP [28]	MDE [27]	SADE_ALM [26]	ABC
*P*_*g*1_ (MW)	50	200	149.7331	197.426	193.2903	199.5669
*P*_*g*2_ (MW)	20	80	52.0571	52.037	52.5735	20.0000
*P*_*g*5_ (MW)	15	50	23.2008	15.000	17.5458	20.7229
*P*_*g*8_ (MW)	10	35	33.4150	10.000	10.0000	21.9065
*P*_*g*11_ (MW)	10	30	16.5523	10.001	10.0000	18.4791
*P*_*g*13_ (MW)	12	40	16.0875	12.000	12.0000	13.5253
Loss (MW)			7.6458	13.064	12.0096	10.8007
Min. cost ($/h)			953.573	930.793	944.031	923.8449
Avg. cost ($/h)			956.460	—	—	940.9169
Max. cost ($/h)			958.263	—	—	1001.0445
Std. dev.			1.720	—	—	13.5609
CPU (s)			93.583 (min.)	41.85	16.160 (min.)	33.51
Adjusted CPU (s)			56.38 (min.)	37.82	13.63 (min.)	33.51

**Table 3:** Result comparison for the IEEE 57-bus system.

	Min	Max	HGA [30]	QN [29]	ACO [29]	ABC
*P*_*g*1_ (MW)	0	575.88	266.850	275.41	242.89	213.7302
*P*_*g*2_ (MW)	0	100	100	98.95	95.05	160.0978
*P*_*g*3_ (MW)	0	140	140	137.75	138.89	140.0000
*P*_*g*6_ (MW)	0	100	100	99.27	97.87	95.9463
*P*_*g*8_ (MW)	0	550	280.438	289.97	311.02	320.7710
*P*_*g*9_ (MW)	0	100	100	99.05	97.84	100.0000
*P*_*g*12_ (MW)	0	410	281.875	267.56	285.10	243.1805
Loss (MW)			18.40	17.16	17.96	22.9256
Min. cost ($/h)			3171.785	3175.506	3172.202	3105.0009
Avg. cost ($/h)			—	—	—	3642.0526
Max. cost ($/h)			—	—	—	5076.5839
Std. dev.			—	—	—	302.1518
CPU (s)			97.75	—	61.01	49.84
Adjusted CPU (s)			—	—	68.73	49.84

**Table 4:** Result comparison for the IEEE 118-bus system.

	IPSO [31]	ABC
Minimum cost ($/h)	145520.0109	138886.5482
Mean cost ($/h)	158596.1725	142233.6067
Maximum cost ($/h)	184686.8248	149145.3604
Power loss (MW)	—	93.7142
Standard deviation ($/h)	9454.4231	2059.9736
CPU (s)	132.233	69.75
Adjusted CPU (s)	163.30	69.75

**Table 5:** Adjusted CPU times for different test systems.

Method	Cores	Clock (GHz)	GFLOPS	CPU (s)	Adjusted CPU (s)
IEEE 30-bus system with quadratic fuel cost functions					
EP [18]	1	0.2	0.8	51.4	3.1
PSO [24]	1	2.4	9.6	77.672	56.15
ACO [25]	2	1.87	14.96	20	22.53
ABC	2	1.66	13.28	22.73	22.73

IEEE 30-bus system with valve-point effects					
IEP [28]	1	2.0	8.0	93.583 (min.)	56.38 (min.)
MDE [27]	1	3.0	12.0	41.85	37.82
SADE_ALM [26]	1	2.8	11.2	16.160 (min.)	13.63 (min.)
ABC	2	1.66	13.28	22.98	22.98

IEEE 57-bus system					
HGA [30]	—	—	—	97.75	—
QN [29]	2	1.87	14.96	—	—
ACO [29]	2	1.87	14.96	61.01	68.73
ABC	2	2.0	13.28	29.85	29.85

IEEE 118-bus system					
IPSO [31]	2	2.1	16.4	132.233	163.30
ABC	2	1.66	13.28	69.748	69.748

## Appendix

The adjusted CPU times of methods for test systems are given in Table 5.
